# Spinal cord ischemia revealed by a Brown-Sequard syndrome and caused by a calcified thoracic disc extrusion with spontaneous regression: a case report and review of the literature

**DOI:** 10.1186/s13256-023-04208-1

**Published:** 2023-11-29

**Authors:** Sonja Petrovic, Nadine Le Forestier, Pierre-François Pradat, Hugues Pascal-Moussellard, Lydia Chougar

**Affiliations:** 1 Diagnostic Imaging Center, Oncology Institute of Vojvodina, Put Dr Goldmana 4, 21204 Sremska Kamenica, Serbia; 2 Département de Neurologie et Centre SLA IdF. Sorbonne, Département.de Recherche : Études des Sciences et Techniques, Hôpital de La Pitié Salpêtrière, Université, AP-HP. Espaces Régional IdF et National de Réflexion Éthique-Maladies Neuro Évolutives, Université Paris Sud/Paris Saclay, Paris, France; 3 Laboratoire d’Imagerie Biomédicale, Sorbonne Université, CNRS, INSERM, Paris, France; 4 Département de Neurologie, APHP, Hôpital Pitié-Salpêtrière, Centre Référent SLA, Paris, France; 5 Northern Ireland Centre for Stratified Medicine, Biomedical Sciences Research Institute Ulster University, Altnagelvin Hospital, Derry/Londonderry, C-TRIC UK; 6 Sorbonne Université, AP-HP, Hôpital de La Pitié Salpêtrière, Département de Chirurgie Orthopédique, 75013 Paris, France; 7 DMU DIAMENT, Department of Neuroradiology, Sorbonne Université, Institut du Cerveau - Paris Brain Institute - ICM, CNRS, Inria, Inserm, AP-HP, Hôpital de La Pitié Salpêtrière, 75013 Paris, France; 8 Centre de NeuroImagerie de Recherche–CENIR, Institut du Cerveau–ICM, Hôpital Pitié-Salpêtrière, 47 Boulevard de L’Hôpital, 75651 Paris Cedex 13, France

**Keywords:** Spinal cord ischemia, Brown-Sequard syndrome, Disc herniation, Case report

## Abstract

**Background:**

Thoracic disc herniation is relatively uncommon, accounting for less than 1% of all spinal herniations. Although most often asymptomatic, they may represent a rare cause of spinal cord ischemia.

**Case report:**

We report the case of a healthy 43-year-old North African male who presented with a Brown-Sequard syndrome revealing a spinal cord ischemia caused by a thoracic disc extrusion. The initial MRI revealed a calcified disc extrusion at the level of T5-T6 without significant spinal cord compression or signal abnormality. A pattern consistent with a medullary ischemia only appeared 48 h later. The patient was treated conservatively with Aspirin and Heparin, which were discontinued later because of a negative cardiovascular work-up. The calcified disc extrusion, which was later recognized as the cause of the ischemia, decreased spontaneously over time and the patient recovered within a few months.

**Conclusions:**

Our case highlights the challenge in diagnosing and managing this uncommon condition. We propose a literature review showing the different therapeutic strategies and their corresponding clinical outcomes.

## Introduction

Spinal cord ischemia is rare, especially in young patients, with an estimated frequency of 0.3–1% of all strokes. Common causes include atherosclerosis, cardioembolism, aortic dissection or surgery [1]. Rare cases of spinal cord ischemia related to disc herniation have been reported in the literature including two cases revealed by a Brown-Sequard syndrome [2, 3]. Differential diagnosis with a compressive myelopathy caused by the disc herniation can be difficult and therapeutic management is controversial. Here we describe a case of a spinal cord ischemia presenting as a Brown-Sequard syndrome secondary to a calcified thoracic disc extrusion that regressed spontaneously over time in a 43-year-old male.

## Case report

A healthy physically active 43-year-old North African male, without relevant medical history except smoking cessation, presented with sudden acute pain in the left lumbar region while jogging. This was accompanied by a rapid onset of motor deficit in the left lower limb and sensory deficit with paresthesia extending from below the umbilical level to the right lower limb. The upper limbs were unaffected, but there was also urinary dysfunction. Upon clinical examination, a severe motor deficit of the left leg was observed, scoring 2/5 on the MRC scale, along with a slight increase in muscular tone based on the modified Asworth's scale. The patellar reflex on the affected extremity was more pronounced, and there was a loss of superficial sensation below the T10 dermatome, involving the right lower limb. The presentation was consistent with a partial Brown-Sequard syndrome.

**Fig. 1:**
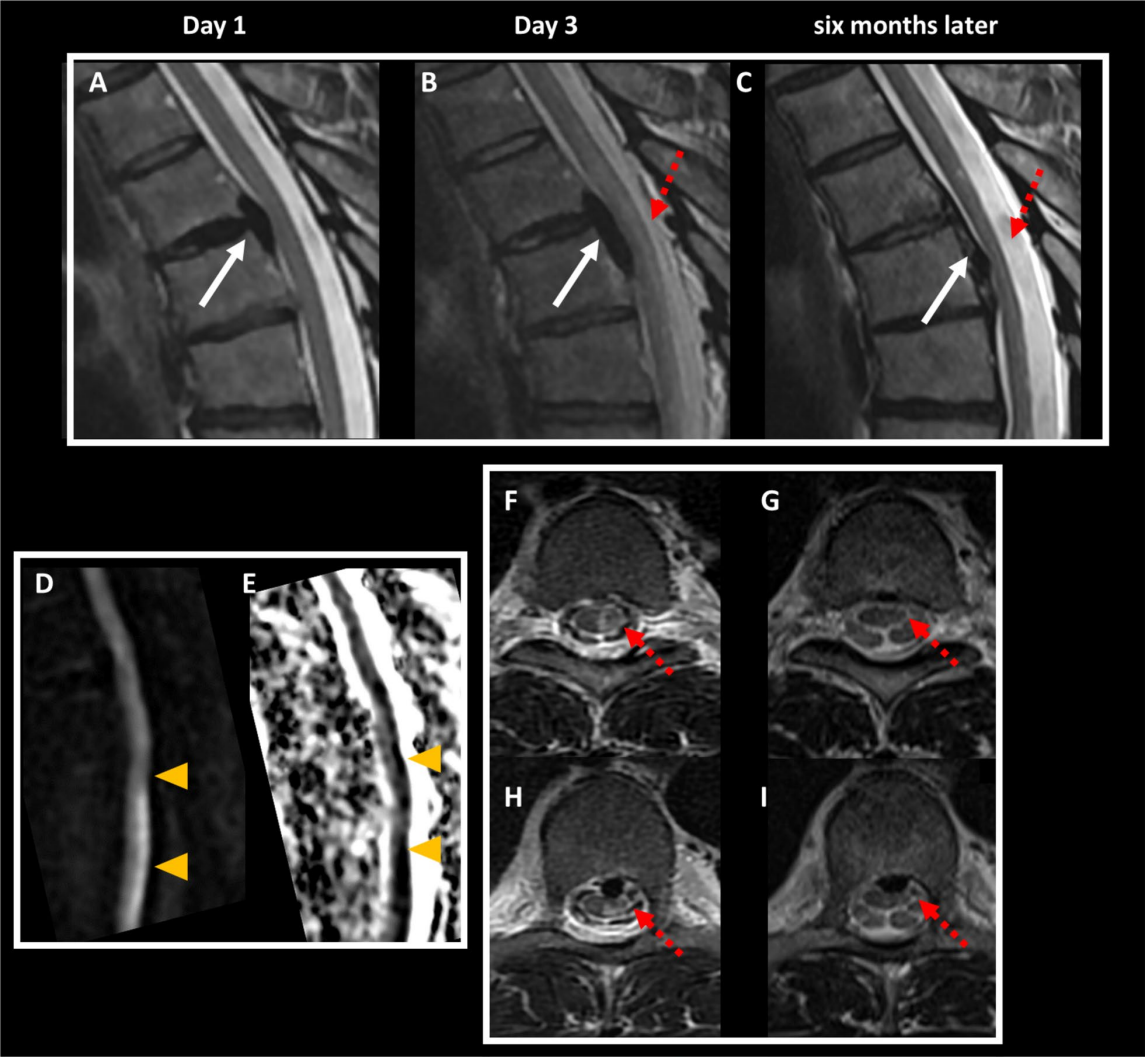
Evolution of MRI findings over time. Initial MRI scan showed a disc extrusion at the T5–T6 level (arrows) without any spinal cord signal abnormality (**A**). The MRI performed 2 days later showed an increase in size of the disk extrusion and the appearance of an extensive spinal cord edema (dashed arrows) (**B**) involving the left lateral half of the cord as seen on axial T2-weighted images (dashed arrows) (**F**, **G**). Diffusion-weighted images revealed a moderate area of hyperintensity (**D**) with a low apparent diffusion coefficient (arrowheads) (**E**) in favor of an ischemic process. Follow-up MRI 6 months later showed a partial regression of the disc herniation (arrows) and a decrease of the signal abnormalities with the appearance of an atrophy (dashed arrows) (**C**, **H**, **I**)

**Fig. 2:**
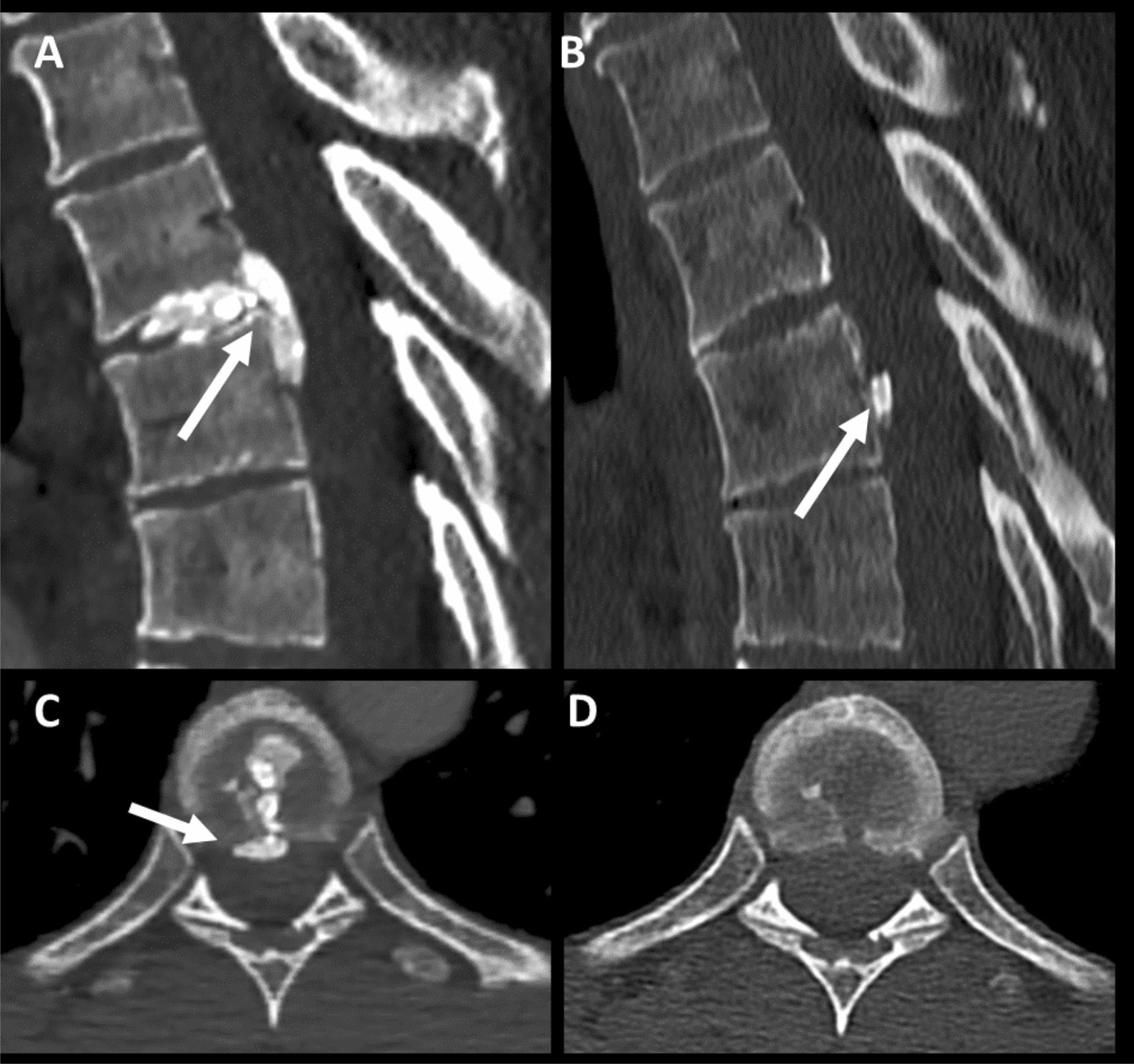
Findings on CT scan. Calcified posteromedial thoracic disc extrusion slightly lateralized to the left at the T5-T6 level with an 18 mm craniocaudal migration (arrows), abutting the anterior aspect of the spinal cord, without significant canal stenosis (**A**, **C**). Six months later, spontaneous decrease of the disc extrusion with a residual calcification adjacent to the posterior wall of T6 (**B**, **D**)

The contrast-enhanced CT of the abdomen and pelvis initially performed was unremarkable. An MRI of the spinal cord was performed 24 h after the onset of symptoms, revealing a posteromedial disc extrusion slightly lateralized to the left at the T5-T6 level with an 18 mm craniocaudal migration, without significant canal stenosis. The disc extrusion abutted the anterior aspect of the spinal cord, with no associated signal abnormalities (Fig. 1). A further CT of the spine confirmed the presence of a calcified disc extrusion in continuity with a disc calcification (Fig. 2). Brain MRI was normal.

The patient was referred to the department of Neurosurgery, where a surgical intervention was not deemed necessary due to the lack of substantial spinal cord compression. The treatment initially consisted of intravenous bolus of corticosteroids based on an inflammatory hypothesis.

Following a worsening of neurological symptoms 48 h later, a second MRI was performed, showing an increase in size of the disc extrusion without significant canal stenosis. Furthermore, a spinal cord edema appeared, with a longitudinal hyperintensity on T2-weighted images extending from T5 to T7 and involving the left lateral half of the cord, mainly in the posterior part. Diffusion-weighted images revealed moderate diffusion restriction of the spinal cord (Fig. 1). CSF analysis was normal. The diagnosis of definitive spontaneous spinal cord ischemia was established based on several factors, including the sudden onset of symptoms, the rapid clinical evolution, the specific imaging pattern including the lesion distribution, the restricted diffusion and the absence of significant spinal cord compression by the disk calcification, and the exclusion of alternative diagnoses [4].

At this stage, a multidisciplinary staff meeting involving a neurologist, neurosurgeon, and radiologist was conducted. Considering the absence of significant spinal cord compression attributable to the calcification and the confirmed diagnosis of medullary ischemia, a consensus was reached to refrain from surgical intervention and a conservative treatment approach was pursued. Treatment by oral Aspirin (100 mg per day) and subcutaneous injection of Heparin (4000 UI per day) was initiated. Aspirin was rapidly discontinued in the absence of atherosclerosis or dissection of the aorta or its branches on the CT angiography. The cardiac workup was normal. The disc extrusion was later recognized as the cause of the infarction. Additional tests including antiphospholipid antibodies testing were negative.

Following rehabilitation, the patient showed significant improvement in his clinical condition. Subsequent examinations revealed an enhancement in motor deficit, with the left leg now scoring 4/5 on the MRC scale. The patient recovered around 70% of his motor function within a few months. However, he reported persisting feelings of stiffness in the leg. Abnormalities in sensation in the right lower limb, specifically regarding temperature and crude touch, were still present. Urinary function was fully restored. The patient was able to resume his professional activities after 6 months. During the follow-up period, imaging examinations performed at 5 months and one year showed a spontaneous reduction in the size of the calcified posteromedial extrusion, along with calcification within the disc (Figs. 1 and 2). The spinal cord hyperintensity decreased accompanied by the appearance of atrophy (Fig. 1).

## Discussion

Brown-Sequard syndrome results from an hemisection of the spinal cord and is characterized by the association of (i) an ipsilateral loss of motor function due to the involvement of the pyramidal tract, (ii) an ipsilateral loss of tactile discrimination and of vibratory and positional sensation secondary to the interruption of the ascending fibers in the posterior column and (iii) a contralateral loss of pain and temperature sensation resulting from spinothalamic tract dysfunction. The syndrome is more often caused by traumatic medullary injuries and extramedullary spinal cord tumors [5]. Only a few cases of spinal cord ischemia presenting as a Brown-Sequard syndrome were reported in the literature, following dissection of the vertebral arteries at the cervical level [6, 7] or arterial compression by disc herniation at the thoracic level [2, 3]. In our patient, the disc herniation was later recognized as the underlying cause of the ischemia, following a negative vascular workup. Acute compressive myelopathy secondary to the herniation was the main differential diagnosis. However, the ischemic hypothesis was favored due to several factors, including the absent of significant compression of the spinal cord, the signal abnormalities extending beyond the herniation level in a vascular distribution pattern, and the presence of diffusion restriction on MRI. Our case also serves as a reminder that a negative initial MRI should not definitively exclude the possibility of ischemia and that repeat imaging within 24 h may be necessary.

The spinal cord is supplied by an unpaired anterior midline vessel called the anterior spinal artery (ASA) and paired posterior spinal arteries. Radicular branches feed both anterior and posterior spinal arteries in a metameric fashion. Intrinsically, the spinal cord is fed by sulco-commissural arteries originating from the ASA in a centrifugal fashion. Each sulco-commissural artery supplies half of the cord [8]. The ASA is more commonly involved, resulting in an ASA syndrome. The posterior spinal artery syndrome is relatively infrequent, while total transverse spinal cord ischemia involving both anterior and posterior spinal arteries has rarely been described. Brown-Sequard syndromes are thought to arise from the involvement of a single sulco-commissural artery [2].

In our case, two concomitant mechanisms may have led to the development of ischemia. The herniation, which was slightly lateralized on the left, could have compressed and/or stretched the adjacent left sulco-commissural artery, causing a hypoperfusion in its territory. On the other hand, a fibrocartilaginous embolism (FCE) could have occurred following the rupture of the nucleus pulposus. FCE is a rare cause of spinal cord and cerebral ischemia, whose definite diagnosis relies on autopsy. Most cases of FCE have been found associated with physical exertion, minor trauma or Valsalva maneuver before severe spinal cord infarction [2, 9]. In our case, the physical exercise preceding the symptom onset likely caused the rupture of the nucleus pulposus and subsequent herniation. This herniation was responsible for the lumbar pain, and is believed to have triggered the vascular events that ultimately resulted in the ischemic condition.

Treatment of symptomatic calcified disc herniation, especially when associated with ischemia, remains controversial. Some authors recommend surgery in case of symptom progression or in the absence of improvement after conservative treatment. Our patient was treated conservatively with favorable clinical evolution and spontaneous regression of the calcified extrusion over time, although a sequelae atrophy of the spinal cord appeared. To our knowledge, eleven cases of spinal cord ischemia related to thoracic disc herniation have been reported, including only two cases of Brown-Sequard [2, 3]. Therapeutic management was heterogeneous with two cases treated with surgery [8, 10] and seven cases managed conservatively [2, 11–15], with variable clinical outcome (Table 1).

**Table 1:** Cases of spinal cord ischemia caused by thoracic disc herniation reported in the literature: therapeutic management and clinical outcome

Study	Age (years)	Sex (F/M)	Level	Calcified herniation	Spinal artery syndrome	Initial presentation	Surgery	Medical treatment	Clinical outcome
Aalbers [8]	54	F	T8–9	Yes	Posterior	Paresthesia and paresis of the left leg (4/5)	Yes	Corticosteroids	Walk short distances with a walker
Santillan [10]	54	F	T8–9	No	Anterior	Severe lower extremity weakness, sensory deficit below T3, impaired proprioception, saddle anesthesia,	Yes	Nm	Complete
Astreinidis [2]	45	F	T8–9	No	Brown-Sequard	Severe paraparesis (1–2/5)	No	Corticosteroids, Heparin	Walk small distances without a walker after 3 months, no improvement after 6 months; spontaneous regression of the herniation
Chiche [12]	40	F	T6–7, T7–8	Yes	Anterior	Paraesthesia and paraplegia below T4	No	Nm	Partial recovery with a severe residual deficit after 3 months
Chiche [12]	57	F	T10–11	Nm	Anterior	Paraparesis below T10	No	Nm	Partial recovery with a severe residual deficit after 3 months
Reynolds [15]	36	F	T7–8	No	Anterior	Paraplegia, sensory deficit	No	Nm	Near complete motor recovery with residual decreased sensation after 8 months
Lopez-Gonzales [13]	45	F	T8–9	Yes	Anterior	Complete paraplegia and sensory loss below T8-9 level	No	Corticosteroids	No recovery
Dolan [14]	36	F	T6–7	Yes	Brown-Sequard	Right paraparesis (1–2/5), numbness, proprioception, vibration loss	No	Nm	Mild residual weakness of the right limb and numbness of the left foot after two years
Yano 2003 [16]	78	M	T8–9	Unknown	Anterior	Unknown	Unknown	Nm	Unknown
Guest 2000 [11]	38	F	T8–9	No	Anterior	Severe paraparesis (1–2/5)	No	Corticosteroids, aspirin	Recovery after 2 weeks
Mansour [3]	49	F	T9–10	Yes	Brown-Sequard	Unknown	Unknown	Nm	Unknown

*F* female, *M* male, *nm* not mentioned

## Conclusion

We report a rare case of spinal cord ischemia revealed by a Brown-Sequard syndrome, secondary to a calcified thoracic disc extrusion in a healthy 43-year-old male. The evolution under conservative treatment was favorable with spontaneous regression of the disc extrusion and neurological recovery within a few months. The case underscores the challenges associated with the diagnosis and therapeutic management of this particular pathology. Studies on large series are needed to provide standardized therapeutic guidelines.

## Data Availability

Not applicable.

## Abbreviations

ASA: Anterior spinal artery
FCE: Fibrocartilaginous embolism
